# Prompting large language models to extract chemical‒disease relation precisely and comprehensively at the document level: an evaluation study

**DOI:** 10.1371/journal.pone.0320123

**Published:** 2025-04-08

**Authors:** Mei Chen, Tingting Zhang, Shibin Wang

**Affiliations:** 1 Key Laboratory of Ethnic Language Intelligent Analysis and Security Governance of MOE, Minzu University of China, Beijing 100081, China; 2 School of Information Engineering, Minzu University of China, Beijing 100081, China; IBM Research - Israel, ISRAEL

## Abstract

Given the scarcity of annotated data, current deep learning methods face challenges in the field of document-level chemical-disease relation extraction, making it difficult to achieve precise relation extraction capable of identifying relation types and comprehensive extraction tasks that identify relation-related factors. This study tests the abilities of three large language models (LLMs), GPT3.5, GPT4.0, and Claude-opus, to perform precise and comprehensive extraction in document-level chemical-disease relation extraction on a self-constructed dataset. Firstly, based on the task characteristics, this study designs six workflows for precise extraction and five workflows for comprehensive extraction using prompting engineering strategies. The characteristics of the extraction process are analyzed through the performance differences under different workflows. Secondly, this study analyzes the content bias in LLMs extraction by examining the extraction effectiveness of different workflows on different types of content. Finally, this study analyzes the error characteristics of extracting incorrect examples by the LLMs. The experimental results show that: (1) The LLMs demonstrate good extraction capabilities, achieving the highest F1 scores of 87% and 73% respectively in the tasks of precise extraction and comprehensive extraction; (2) In the extraction process, the LLMs exhibit a certain degree of stubbornness, with limited effectiveness of prompting engineering strategies; (3) In terms of extraction content, the LLMs show a content bias, with stronger abilities to identify positive relations such as induction and acceleration; (4) The essence of extraction errors lies in the LLMs’ misunderstanding of the implicit meanings in biomedical texts. This study provides practical workflows for precise and comprehensive extraction of document-level chemical-disease relations and also indicates that optimizing training data is the key to building more efficient and accurate extraction methods in the future.

## Introduction

The relationship between chemicals and diseases is highly complex. For example, aspirin decreases the risk of heart disease and stroke, while it also increases the risk of gastrointestinal ulcers and bleeding. Grapefruit notably amplifies both the therapeutic effects and side effects of statins. Identifying the relationships between chemicals and diseases precisely and comprehensively constitutes a fundamental task in biomedical research. Since the beginning of this century, numerous chemical‒disease relationships have been discovered and recorded in the academic literature [1–5]. Consequently, the automatic extraction of these relationships has emerged as a focal topic in scientific research [6–10]. Using automatic extraction techniques, scientists can efficiently analyze the complex relationships embedded within the expansive literature, thereby accelerating the progress of precision medicine and drug development.

Owing to the complexity of the chemical‒disease relationship, a single sentence often fails to encapsulate its full scope, necessitating multiple sentences for comprehensive expression [11]. Consequently, extracting chemical‒disease relationships at the document level is highly important. In recent years, with the advancement of deep learning techniques, the extraction of chemical‒disease relationships at the document level has undergone rapid development. In the field of dataset construction, scientists have developed a specialized chemical‒disease relation (CDR) dataset [12]. This dataset contains 1500 abstracts from papers, each with a co-occurrence relation annotation between chemical and disease. The CDR dataset is highly authoritative, with chemical‒disease relationships in other biomedical datasets [13] being derived from the CDR [12]. Concerning extraction methodologies, scientists have established methods such as seq2seq [14] and the supervised augmented intermediate step SAIS [15], which achieve F1 scores of 67.2% and 79% on the CDR dataset, respectively. In 2024, the innovative Bio-DocRE framework HTGRS [16] achieved an F1 score of 86.9% on the CDR dataset by integrating key information sources in the document through a hierarchical tree graph and enhancing relation reasoning based on the entity pair using a relation segmentation module.

**Fig 1:**
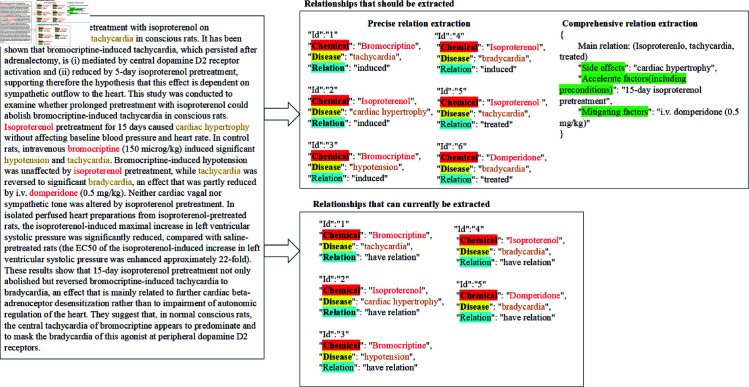
Diagram of current shortcomings in document-level chemical-disease relation extraction. The left-hand text covers specific types of relationships between chemicals and diseases, in addition to related side effects and factors that influence these relationships. However, current research is limited to determining whether there is an association between chemicals and diseases.

Despite these advancements, current extraction methods still do not meet the academic community’s expectations for identifying chemical‒disease relationships, primarily because deep learning-based models require vast quantities of high-quality annotated data. This limitation is reflected in the following aspects (as shown in Fig 1): (1) the extracted relations are ambiguous [17]. The existing CDR dataset only annotates the co-occurrence relation, merely recording the presence of a relation without clarifying whether it pertains to “induced” or “treated.” Therefore, current deep learning models can extract only co-occurrence relations and cannot reflect actual relations such as “induced” and “treated.” (2) The extracted relations are one-sided [18]. The relationships between chemicals and diseases are highly complex and rarely conform to simple binary structures. For example, a single chemical might induce multiple diseases, while a single disease can be treated by several different chemicals. Additionally, “induced” or “treated” processes are influenced by factors such as temperature, pH, and other chemicals. This information remains unannotated in the current CDR dataset, which prevents existing deep-learning models from performing comprehensive relation extraction. Considering the intricate nature of chemical‒disease relationships, the annotation process requires expert involvement and is inherently time-consuming and labor-intensive. Therefore, there is an urgent need within the field to develop extraction methods that reduce the reliance on annotation.

In the realm of artificial intelligence, large language models (LLMs) [19–24] alongside prompt engineering [25–32] have emerged as pivotal tools in propelling the advancement of natural language processing. Prompt engineering can fully utilize the strong reading comprehension abilities and rich world knowledge that LLMs possess to demonstrate learning ability without the need for annotated data, known as zero-shot learning, to provide strong support for the implementation of various tasks. In this study, to address the issues of ambiguity and one-sidedness present in existing research, we evaluate the capabilities of LLMs—GPT-3.5, GPT-4.0, and Claude-opus—in two scenarios: precise extraction and comprehensive extraction, across both the extraction processes and content on a self-constructed dataset. We designed six precise extraction workflows and five comprehensive extraction workflows based on various prompt engineering strategies, and the characteristics of the extraction process and content biases of LLMs were elucidated by assessing the differences in results under different workflows. Ultimately, based on the empirical data derived from these tests, we summarized the cause of errors observed in the extractions conducted by LLMs.

## Materials and methods

### The evaluation methods of precise relation extraction

**Fig 2:**
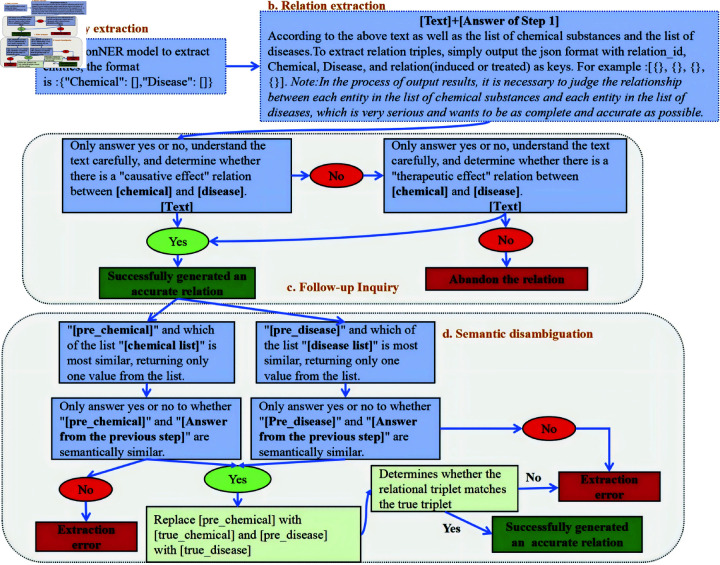
Flowcharts for precise relation extraction.

The precise relation extraction task aims to extract chemical-disease entity pairs from documents and accurately identify their relationships (“induced” or “treated”). Based on task characteristics and prompt engineering strategies, a detailed workflow comprising four independent modules has been designed, as illustrated in Fig 2. By combining different modules from these modules, various workflows can be formed. The descriptions of each module are as follows:

(a) Entity extraction: This phase involves the thorough extraction of all chemical and disease entities from the literature, ensuring minimal interference from unrelated content during relation extraction. This module can be implemented using two approaches: (1) Extraction using LLMs; (2) Extraction via the pre-trained model, ConNER [33].
(b) Relation extraction: The primary objective of this module is the extraction of chemical-disease relationships. To achieve this and constrain model responses, we utilize two distinct methodologies: One approach entails mapping each chemical entity, as identified in the entity list from the stage (a), to all corresponding disease entities, facilitated through Note_prompt. The alternative method employs a direct extraction approach and does not utilize Note_prompt.
(c) Follow-up inquiry: This stage reduces the hallucination of LLMs by requiring models to reflect on the relations identified in the preceding phase, with responses limited to “yes” or “no.” If the LLMs answers “no,” a further determination is required to ascertain whether an alternative relation exists.
(d) Semantic disambiguation: The synonymy of terms is a prevalent phenomenon in the biomedical literature. For example, within a scholarly article, the terms “chronic liver disease” and “liver disease” often denote the same disease, and “high blood pressure” might be indicated by the symptom “dizziness.” To circumvent errors stemming from synonymy, this step uses the world knowledge embedded in LLMs for semantic disambiguation. In this stage, only the LLM is allowed to answer “Yes” or “No” to facilitate subsequent processing.

### The evaluation methods of comprehensive relation extraction

The comprehensive relation extraction task aims to extract information on chemical-disease relationships from documents, including (1) Side effects during the treatment process. (2) Accelerating factors that enhance the development of the relationship, including any preconditions. (3) Mitigating factors for the relationship.

**Fig 3:**
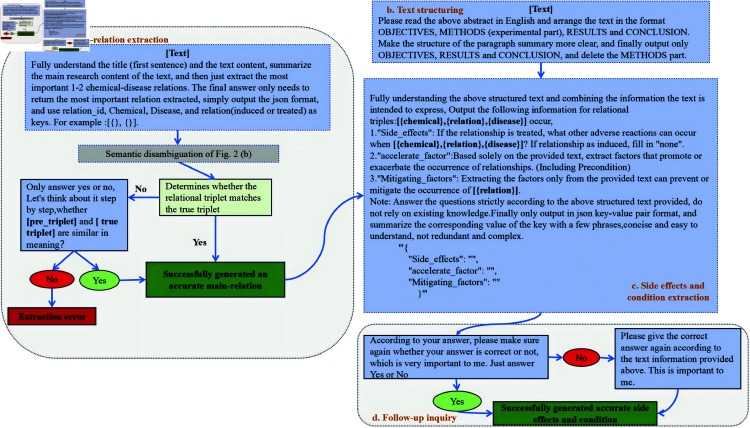
Flowcharts for comprehensive relation extraction.

Similarly to precise extraction, a detailed workflow consisting of four independent modules has been designed, as shown in Fig 3. Likewise, various workflows can be created by combining different modules. The descriptions of each module are as follows:

(1) Main relation extraction: This step directly extracts predominant relations, which serve as the research objects, from the abstract to provide a foundation for subsequent comprehensive relation extraction, and these relations undergo a rigorous semantic disambiguation process, as depicted in Fig 2.
(2) Text structuring: This step restructures the abstract into a clearly defined format that encompasses objectives, results, and conclusions, thereby simplifying comprehension of LLMs. This approach facilitates the removal of superfluous information, emphasizing critical content and enhancing the LLMs’ ability to capture significant information.
(3) Side effects and condition extraction: Based on the main relations extracted, this phase focuses on extracting the side effects and conditions that influence the main relation. To prevent the answers of the LLM from deviating from the text, the output content is too cumbersome and inconvenient to evaluate. We designed a Note prompt to constrain model responses that must provide thorough and succinct responses based on the text, output is in JSON format, ensuring that the extracted conditions are exhaustive and accurate. This module utilizes two types of prompts: one including a Note_prompt and one without.
(4) Follow-up inquiry: To mitigate hallucinations in LLMs, this step requires the models to engage in reflection, thereby ensuring the validation of previously extracted side effects and conditions. A rigorous binary (“yes” or “no”) response protocol is applied, and the emotional statement “which is very important to me,” which can help the model focus on specific key information [34], is used to enhance the targeting and accuracy of information extraction. If the LLM answers “no,” it is then prompted to refine its answer based on a deeper understanding.

By executing different combinations of modules within the aforementioned workflow, we tested the extraction effects and biases during extraction, and based on the test results, we analyzed the characteristics of LLM extraction of chemical-disease relationships.

### Datasets

The datasets used in this study were constructed based on the existing BC5CDR dataset [12]. TeamTat [35] and PubTator [36] were used for annotation. The annotation rules of the BioRed dataset[13] are adopted for annotation. All of the above data annotations were performed via the LLMs GPT4-Web version to assist in understanding the data and to improve the efficiency and accuracy of the annotations. The resulting dataset consists of 200 PubMed articles with 1695 annotated chemicals, 1632 diseases, and 778 chemical‒disease relationships. Of these, there are 219 treated relations and 559 induced relations. Additionally, 100 pieces of data were annotated for comprehensive relation extraction, including 12 side effects, 105 acceleration factors, and 38 mitigation factors.

### Model setup

The OpenAI ChatGPT and Claude APIs were utilized within Python 3.10.6. Specifically, models gpt-3.5-turbo-0125, gpt-4-0125-preview, and Claude-3-opus were employed, and the following model parameters were set: the temperature was set to 0.0, and the remainder were configured to their default values.

### Evaluation metrics

#### Evaluation metrics for precise relation extraction.

The main metrics used to evaluate the effectiveness of this research are precision Eq. (1), recall Eq. (2), and the F1 score Eq. (3), which are defined as follows:


$$\begin{array}{ccc} \text{Precision} & =\frac{\text{True Positive}}{\text{True Positive}+\text{False Positive}} & \end{array}$$ (1)

$$\begin{array}{ccc} \text{Recall} & =\frac{\text{True Positive}}{\text{True Positive}+\text{False Negative}} & \end{array}$$ (2)

$$\begin{array}{ccc} F1 & =\frac{2\times (\text{Precision}\cdot \text{Recall})}{\text{Precision}+\text{Recall}} & \end{array}$$ (3)

In this study, triples manually extracted and annotated serve as the ground truth. Each triple extracted by the LLMs is compared with every triple in the ground truth. If it is equivalent to a triple in the ground truth, it is categorized as a true positive; if it is not equivalent to any triple in the ground truth, then it is counted as a false positive. All extracted triples are merged according to their MESH value pairs. The number of false negatives is determined by subtracting the number of true positives from the total number of annotated triples.

In the preceding description, “equivalent triples” are defined as follows: if the chemical entity and the disease entity in two triples have the same MESH values, and if the relation types are identical for both triples, then the two triples are considered equivalent. For example, the triples (“vigabatrin,” “induced,” “visual field loss”) and (“vigabatrin,” “induced,” “visual field constriction”) are considered equivalent, as both involve the same chemical entity (MESH: D020888) and the same disease entity (MESH: D014786) with the relation type “induced.” Despite the different disease names used in the triples, they are recognized as equivalent.

#### Evaluation metrics for comprehensive relation extraction.

For this task, the BERTS score was selected for evaluation [37]. The BERTScore was designed to automatically compute the precision, recall, and F1 score by calculating the cosine similarity between two sentences. The evaluation based on similarity is more in line with the linguistic complexity of the results of comprehensive extraction.

#### Entropy calculation.

To measure the information complexity of each abstract text, Shannon entropy is employed to characterize the amount of information within the abstract. For a text containing multiple characters, the information entropy H(X) Eq. (4) is calculated based on the probability of occurrence of each character in the text. The formula is as follows:


H(X)=−∑i=1np(xi)log ⁡ 2(p(xi)) (4)

wherein H(X) represents the entropy of the text, $x_{i}$ is the i-th distinct character in the text, and p($x_{i}$) Eq. (5) is the probability of occurrence of the character. This probability is calculated as the number of times the character appears in the text divided by the total number of characters N in the text, expressed as:


$$\begin{array}{ccc} p(x_{i})=\frac{n_{i}}{N} & & \end{array}$$ (5)

where $n_{i}$ is the frequency of occurrence of the character $x_{i}$ in each abstract text, N is the total number of characters in that abstract text, and the unit of entropy is bits.

In this study, we first count the characters in each abstract, including letters, numbers, spaces, and punctuation marks, and calculate the frequency of occurrence for each character. Subsequently, we divide the frequency of each character by the total number of characters in the abstract to obtain the probability of occurrence for each character. Then, using the formula for Shannon entropy Eq. (4), we calculate the contribution of each character to the total entropy. Finally, we sum the entropy contributions of all characters to obtain the overall entropy of the abstract. The entropy value reflects the complexity of the character distribution within the abstract; a higher value indicates a more uniform distribution of characters and greater uncertainty in the information.

To compare the differences in entropy between two groups of abstracts (high F1-score abstracts and low F1-score abstracts), we employed the independent samples T-test. The independent samples T-test assumes that the data from the two groups come from different populations and compares their means. The null hypothesis ($H_{0}$) posits no significant difference in the mean entropy between abstracts with high F1 scores and those with low F1 scores. The alternative hypothesis ($H_{1}$) asserts a significant difference in the mean entropy between these groups. The formula for the independent samples T-test Eq. (6) is as follows:


$$\begin{array}{ccc} t=\frac{{\bar{X}}_{1}-{\bar{X}}_{2}}{\sqrt{\frac{s_{1}^{2}}{n_{1}}+\frac{s_{2}^{2}}{n_{2}}}} & & \end{array}$$ (6)

where ${\bar{X}}_{1}$ and ${\bar{X}}_{2}$ represent the sample means of the two groups,$s_{1}^{2}$ and $s_{2}^{2}$ are the sample variances, $n_{1}$ and $n_{2}$ are the sample sizes of the two groups. The denominator is the square root of the pooled standard error of the two samples.

The computed T-value reflects the magnitude of the difference between the two means relative to the variability within the data. Subsequently, the T-value and degrees of freedom are used to find the corresponding p-value, which determines whether to accept or reject the null hypothesis. If the p-value is less than the set significance level (0.05), the difference between the two means is considered statistically significant.

## Results and discussion

### Evaluation of precise relation extraction

**Fig 4:**
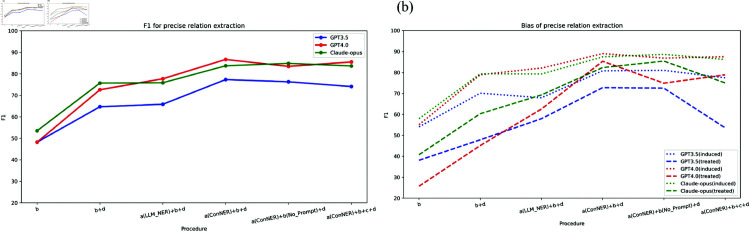
Precise relation extraction results. (a) the overall results of GPT 3.5, GPT 4.0, and Claude-opus in different workflows; (b) the bias results of GPT 3.5, GPT 4.0, and Claude-opus in different workflows; “a” represents Entity extraction, “b” represents Relation extraction, “c” represents Follow-up inquiry, and “d” represents semantic disambiguation.

We conducted tests on six different workflows. The test results are shown in Fig 4, and the detailed analysis is as follows.

#### Analysis of extraction effect

As depicted in Fig 4a, executing solely the relation extraction step b results in suboptimal performance across all three models, with F1 scores of approximately 50%. By adjusting different modules in the workflow, we can effectively improve the results. The analysis of the results leads to the following conclusions:

(1) After adding the semantic disambiguation module (b+d), the F1 values of all three models increased significantly by about 20%. This indicates that the world knowledge of the LLM can effectively perform disambiguation tasks in this domain.
(2) After incorporating the LLM’s entity extraction step (LLM_NER+b+d), the F1 values of the three models showed no significant change. This suggests that splitting relation extraction into two steps—entity extraction and relation extraction—does not noticeably enhance the results. The LLM follows its inherent patterns during relation extraction, and specifying extraction steps does not alter its fundamental extraction approach.
(3) After adding the traditional model ConNER for entity extraction (ConNER+b+d), the F1 values of the three models improved by about 10% from the b+d baseline, reaching or nearing the highest F1 values. This is because the traditional model ConNER extracts biomedical entities more efficiently than the LLM, providing a high-quality list of entities. This highlights the importance of high-quality entity recognition about extraction. Additionally, removing the Note_prompt in relation extraction resulted in no significant changes in the F1 values of the models, indicating that requiring the model to establish one-to-one correspondence for entities does not have a noticeable impact on the results. It is again proved that relation extraction of LLMs follows an inherent pattern, and artificial steps are of little significance.
(4) After adding the follow-up Inquiry step, there were no significant changes in the F1 values of the models, suggesting that the models are quite confident in their extraction results.

In summary, in our tests, by establishing appropriate workflows, three LLMs all achieved commendable results in precise extraction tasks. Among these, the GPT-4 model reached the highest F1 score of 87%, demonstrating a strong comprehension of chemical-disease relationship types. This indicates substantial potential for the application of LLMs in this field.

#### Analysis of bias.

Fig 4b reveals that by adjusting different modules in the workflow, all three LLMs consistently demonstrate superior extraction performance for “induced” relationships over “treated” relationships. This observation suggests an inherent bias in relation extraction towards certain types of relationships within LLMs. Based on the analysis of the results, the following conclusions can be drawn:

(1) LLMs exhibit a markedly enhanced extraction capability for “induced” relationships when compared to “treated” relationships. Initially, across various workflows, the F1 score fluctuations for “induced” relationships in the three models are significantly less pronounced than those observed for “treated” relationships. The range between the maximum and minimum F1 scores for “induced” relationships is approximately half that for “treated” relationships, indicating that the models are more consistent in extracting “induced” relationships. Furthermore, the incorporation of follow-up inquiry leaves the F1 scores for “induced” relationships largely unaffected, whereas those for “treated” relationships exhibit a discernible decrease. This suggests that the models are less certain about “treated” relationships compared to “induced” relationships.
(2) Low-quality entities may compromise the model’s comprehension of relationship types, particularly for “treated” relationships. Furnishing a high-quality entity list can significantly mitigate bias. Following entity extraction using the traditional model ConNER (ConNER+b+d), the F1 scores for “treated” relationships demonstrate substantial improvements across all three models, thereby minimizing the bias discrepancy. This indicates that the difference in understanding between the two relationship types is not significant, especially for more capable models. Providing a high-quality entity list enables the models to focus on relationship types, leading to better performance.
(3) Among the three models, The Claude-opus model exhibits superior balance across relationship types, whereas GPT-4 displays greater variability in performance for “treated” relationships, possessing the most pronounced bias gap among the models.

In summary, we can conclude that LLMs exhibit certain biases. This bias can be mitigated to some extent by adjusting the design of the workflow.

### Evaluation of comprehensive relation extraction

**Fig 5:**
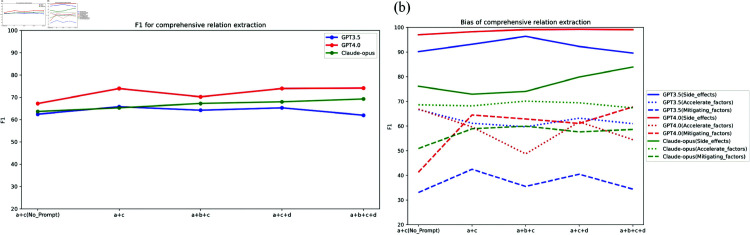
Comprehensive relation results. (a) the overall results of GPT 3.5, GPT 4.0, and Claude-opus in different workflows; (b) the bias results of GPT 3.5, GPT 4.0, and Claude-opus in different workflows; “a” represents Main relation extraction, “b” represents Text structuring, “c” represents Side effects and condition extraction, and "d" represents Follow-up inquiry.

We conducted a test analysis of the effects and biases of the different modules in the workflow. The test results are shown in Fig 5, and the detailed analysis is as follows:

#### Analysis of extraction effect.

Fig 5a illustrates that the comprehensive extraction performance across the three models is remarkably uniform, with GPT-4 exhibiting marginally superior performance relative to the others. Additionally, for each model, the performance differences under workflows composed of different modules are also minimal. Text structuring, follow-up inquiry, and the Note_prompt that requires strict adherence to the text content do not significantly influence the outcomes.

In this section, the highest F1 score achieved was 74% using the A+C workflow on the GPT-4 model. We consider this result to be somewhat unsatisfactory; therefore, we introduced a chain of thought (CoT) in this scenario (“Let’s think step by step”). After incorporating the CoT, the F1 score slightly decreased to 73%, indicating that, similar to precise extraction, comprehensive extraction by LLMs also adheres to their inherent patterns.

In summary, we can conclude that the LLMs exhibit robust understanding capabilities in comprehensive extraction, yielding consistent results across diverse module combinations.

#### Analysis of bias.

It is worth noting that according to the results in Fig 5b, the three models show significant differences in performance across different extraction content. Furthermore, under various extraction contents, the three models also exhibit notable differences in performance within workflows composed of different modules. This indicates that the results shown in Fig 5a are merely superficial. From the detailed analysis of the results in Fig 5b, the following conclusions can be drawn:

(1) LLMs demonstrate a pronounced proficiency in extracting side effects. All three models consistently outperform in the extraction of side effects compared to other contents, revealing an inherent bias toward side effects, attributed to their more distinct and readily extractable presentation in the text.
(2) In the extraction of accelerating and mitigating factors, both Claude-opus and GPT-3.5 exhibit substantially better performance in extracting accelerating factors over mitigating factors, suggesting a predisposition towards accelerating factors. This phenomenon likely stems from the training data for LLMs, which predominately comprise medical literature and texts where descriptions of disease onset, progression, and exacerbation are more prevalent than those of alleviation and mitigation. This data imbalance leads to the model being more sensitive and accurate in extracting conditions like “worsening ... certain diseases” or “increasing ... risk.” Additionally, the F1 scores do not vary much across different workflows, indicating that LLMs have an inherent extraction pattern and are less influenced by instructions.
(3) For the extraction of accelerating and mitigating factors, GPT-4 exhibits considerable fluctuation. In the a+c (No prompt) workflow, GPT-4’s F1 score for accelerating factors is about 20% higher than for mitigating factors. After adding the Note_prompt (a+c workflow), GPT-4’s F1 score for accelerating factors drops significantly, while its F1 score for mitigating factors rises substantially, exceeding the F1 score for accelerating factors by approximately 4%. Text structuring (a+b+c workflow) results in a significant decline in performance for accelerating factors, with an F1 score decrease of about 9%, but has little impact on mitigating factors.
(4) Under various workflows, the Claude-opus model exhibits minimal variation and a small range of differences in extraction performance across different scenarios. Therefore, Claude-opus exhibits commendable data equilibrium.
(5) LLMs display considerable confidence in their results, and the inclusion of follow-up inquiry generally has minimal impact under various conditions.

In summary, we can conclude that a notable bias towards the “accelerate factor” is observed under relative conditions. This bias can be partially alleviated by adding a “Note_prompt.” In addition, The extraction performance of GPT-4 under relative conditions is markedly unstable, whereas Claude-opus demonstrates comparatively stable performance.

### Error analysis

#### The relationship between entropy and extraction effect.

In our study, we first calculated the entropy values of abstracts to analyze the relationship between information complexity (entropy) and extraction performance (measured by the F1 Score). Information entropy reflects the uniformity of character distribution in the text, with higher entropy indicating greater information complexity. Theoretically, information entropy could affect the readability and structure of abstracts, thereby influencing the extraction results.

**Fig 6:**
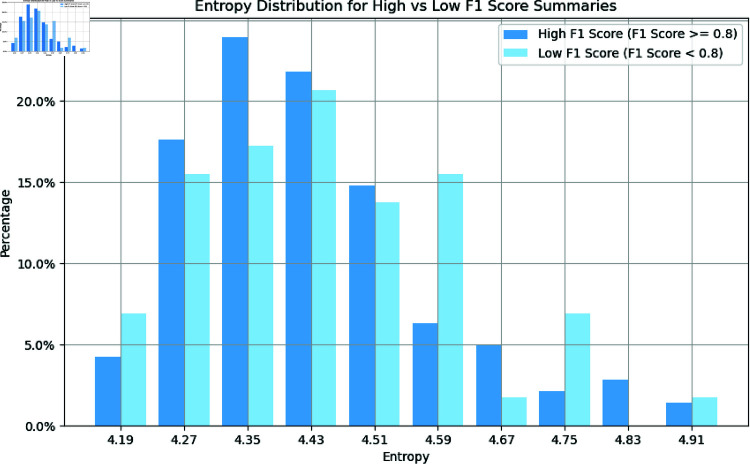
The relationship between model extraction effect and entropy.

For this purpose, we classified the abstracts based on their F1 Scores, calculating the entropy distributions for high F1 Score abstracts (F1 Score 60*%*0.8) and low F1 Score abstracts (F1 Score 60*%*0.8), and plotted a comparison of the entropy distributions (as shown in Fig 6). The graph indicates that the entropy values for both high and low F1 Score abstracts range between 4.19 and 4.91. Although the high F1 Score abstracts tend to cluster around 4.35 to 4.43, the distribution of low F1 Score abstracts is relatively more dispersed. Overall, there is considerable overlap in the entropy distributions of the two categories, and no significant correlation between entropy values and extraction performance is evident.

Further analysis through an independent T-test (p = 0. 23) revealed that the difference in entropy between high and low F1 Score abstracts is not statistically significant. This suggests that information entropy is not a major factor affecting relationship extraction performance. In summary, there appears to be no direct significant association between the entropy of abstracts and their relation extraction outcomes.

#### Statistical analysis for errors.

**Fig 7:**
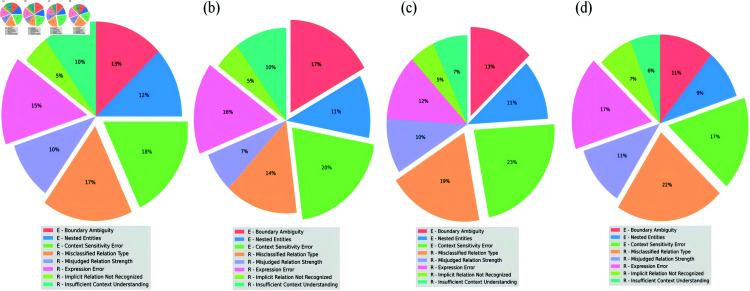
The results of Statistical analysis for errors. (a) the reasons for errors observed in GPT-4.0 data; (b) the factors contributing to incomplete extractions by GPT-4.0; (c) the reasons for errors observed in Claude-opus data; (d) the factors contributing to incomplete extractions by Claude-opus; “E -” represents errors in entity recognition; “R -” represents errors in relation recognition.

Subsequently, we examined the wrong data extracted by LLMs for their precision and comprehensiveness in identifying chemical-disease relationships. As shown in Fig 7, we identified several key factors that contribute to extraction errors.

Through manual analysis of the various types of errors in Fig 7, we found that: Due to the complexity of processes and phenomena involved in biomedical texts, there are diverse forms of hidden meanings within the texts. Although the error forms can be superficially classified into multiple types, they essentially all stem from the LLMs’ inadequate understanding of the hidden meanings in biomedical texts. Here are three typical examples: (1) “Treatment for 2 weeks with Warfarin caused massive focal calcification of the artery media in 20-day-old rats and less extensive focal calcification in 42-day-old rats.” This sentence implies that the focal calcification induced by Warfarin is age-related, but LLMs failed to recognize age as a relevant factor, resulting in a context sensitivity error. (2) “Repeated transient anuria following losartan administration,” In this sentence, LLMs did not realize that adding the modifier "transient" to "anuria" changes the severity of the disease and still treated it as “anuria,” leading to an entity boundary ambiguity error. (3) “The administration of warfarin caused significant bleeding in the patient, and the bleeding was controlled after discontinuing the drug.” In this sentence, the model failed to recognize that “the bleeding was controlled” meant the cessation of bleeding and instead mistakenly interpreted it as the drug discontinuation causing the bleeding, resulting in an expression error.

## Conclusions

In response to the limitations of ambiguity and one-sidedness in existing deep learning methods for document-level chemical-disease relation extraction, this study evaluates the capabilities of LLMs in this domain. The evaluation is bifurcated into precise extraction and comprehensive extraction. The conclusions are as follows:

(1) LLMs have demonstrated substantial potential in the field of document-level chemical-disease relation extraction. Specifically, under the zero-shot learning scenario, through workflow adjustments, LLMs achieved maximum F1 scores of 87% (workflow: Entity extraction via ConNER, followed by Relation extraction, and Semantic disambiguation) and 73% (workflow: Main relation extraction coupled with Side effects and condition extraction) in precise and comprehensive extraction tasks, respectively. In contrast, the method based on deep learning can achieve an F1 score of 86.9% on the CDR dataset for general extraction (i.e., only extracting related chemical and disease entity pairs, without identifying specific relationship types or extracting related conditions). On the BioRED dataset, which contains multiple relationship types, the F1 score is only 66.9%, and it still fails to extract related conditions [16]. This achievement underscores the significant effectiveness of LLMs in information extraction and semantic disambiguation within this domain, leveraging their reading comprehension abilities and inherent world knowledge. Furthermore, the error analysis conducted in this study revealed no significant correlation between extraction accuracy and text entropy, further confirming the robust understanding capabilities of LLMs when processing complex textual information, which is not easily disturbed by textual complexity.
(2) To develop more efficient and accurate methods for document-level chemical-disease relation extraction, we believe the focus of future work lies in optimizing training data. Firstly, the experimental results of this study indicate that LLMs exhibit a certain degree of inflexibility during the extraction process, with limited optimization space for hinting prompt engineering strategies such as detailed steps and self-reflection. This reflects the formation of inherent extraction patterns in LLMs during training, limiting the effectiveness of user instructions. Secondly, at the content extraction level, LLMs demonstrate a bias, particularly in recognizing positive relationships (such as induction and acceleration), which is attributed to the uneven distribution of training data. Finally, the root cause of extraction errors lies in LLMs’ misunderstanding of the implied meanings in biomedical texts. Given the diverse implied meanings embedded in biomedical texts, which is a consequence of the complex processes and phenomena they encompass, there is a pressing need to tailor high-quality training data specifically to bolster LLMs’ comprehension abilities for biomedical texts. To address the aforementioned limitations, future research can be optimized in two main areas: training data sources and training strategies. In terms of data sources, it is recommended to select high-quality data from authoritative biomedical databases such as PubMed, and to carefully design the distribution proportions of the training data to ensure a balanced representation of different types of relationships within the dataset. This will enhance the LLMs’ ability to recognize various relationship types. Regarding training strategies, it is advisable to further explore the model’s decision-making process, and based on its characteristics, select or synthesize representative high-quality examples (including examples of implied meanings) for incorporation into the training dataset. This approach aims to further improve the accuracy and efficiency of LLMs in chemical-disease relation extraction.

This study demonstrates the tremendous potential of LLMs for document-level relation extraction in the biomedical domain. It is important to note that the scope of this work is limited to the extraction of chemical-disease relations. The biomedical field encompasses a variety of complex relationship types, such as the relationships between genes and diseases, each with its own unique characteristics. Given the diversity of relationships in the biomedical domain, future research could focus on exploring how to leverage the extensive world knowledge and powerful reasoning capabilities of LLMs to design specialized and efficient workflows tailored to different types of relations. This direction aims to achieve more accurate and comprehensive document-level relation extraction, thereby advancing the development of information extraction techniques in the biomedical field.

## Supporting information

All data used in this manuscript are provided in open access figshare (10.6084/m9.figshare.26496157).
